# *B. burgdorferi* infection alters synovial macrophage responses in a human *ex vivo* model of Lyme Arthritis

**DOI:** 10.3389/fimmu.2026.1819452

**Published:** 2026-08-18

**Authors:** KariAn E. Mulford, Rachel M. Johnson, Eva S. Oleson, Jessica L. Dennis, Katherine L. Butler, Tyler J. Draayer, Jacob M. Elkins, Christine A. Petersen, Karen I. Cyndari

**Affiliations:** 1 Department of Emergency Medicine, University of Iowa Health Care, Iowa City, IA, United States; 2 Carver College of Medicine, University of Iowa, Iowa City, IA, United States; 3 Department of Epidemiology, College of Public Health, University of Iowa, Iowa City, IA, United States; 4 Interdisciplinary Graduate Program in Immunology, University of Iowa, Iowa City, IA, United States; 5 Department of Orthopedics and Rehabilitation, University of Iowa Health Care, Iowa City, IA, United States; 6 Department of Veterinary Biosciences, College of Veterinary Medicine, The Ohio State University, Columbus, OH, United States

**Keywords:** *B. burgdorferi*, inflammation, Lyme arthritis, Lyme disease, resident synovial macrophage, synovitis, synovium

## Abstract

**Introduction:**

*Borrelia burgdorferi* (Bb) infection can persist in protected sites like articular joints, presenting as migratory oligoarthritis characteristic of Lyme Arthritis (LA). LA is characterized by intermittent synovitis and joint swelling, but why acute infection is relapsing-remitting and not persistent remains unknown. We hypothesize that the immune response is unique to the joint space, particularly the response of Resident Synovial Macrophages (RSMs), and may be important in understanding the clinical course of acute LA and beyond. This study aimed to investigate the polarization and functional profiles of these joint lining macrophages in response to acute Bb exposure in *ex vivo* human synovial tissue from patients undergoing total joint replacement.

**Methods:**

Synovial biopsies from seven patients were exposed to three inoculate doses of Bb (250k, 500k, 1000k) in our Joint Space Analysis System (JSAS). Tissue samples and supernatant were collected at 24, 48, and 72 hours for histology, immunofluorescence (IF), RNAscope, and ELISA.

**Results:**

We found dose- and time-dependent changes in synovial intimal lining (IntL) thickness and cellularity. IntL integrity significantly decreased with 1000k Bb at Day 2 (p = 0.021) and remained decreased with 1000k Bb at Day 3 (p = 0.005). IF revealed sustained expression of the M2 marker CD206 in joint-lining macrophages despite infection. RNAscope demonstrated a spatially limited co-localization of Bb within the tissue and alteration of tight junction ZO-1 subcellular localization within the IntL. ELISA indicated reduced Osteoprotegerin at Day 3, suggesting potential for altered bone remodeling activity. IL-10 secretion increased over time and Bb dose.

**Discussion:**

These findings demonstrate that Bb interaction with joint-lining macrophages is associated with rapid, structural, and functional alterations, with loss of IntL integrity and macrophage dysregulation. Compromise of the synovial barrier may facilitate immune cell infiltration, contributing to disease pathogenesis, in the setting of increased markers of synovitis and increased regulatory cytokines, but causative association of these phenomena remains to be explored.

## 1 Introduction

Lyme Disease (LD), caused by the tick-borne spirochete *Borrelia burgdorferi sensu latu*, is the most common vector-borne illness in the USA. LD affects roughly 476,000 patients annually, a 45% increase from 2010 (1). If treated in the early localized phase, *B. burgdorferi* (Bb) is swiftly eradicated. However, untreated progression leads to hematologic spread and early disseminated LD. Spirochetes may then travel to the brain and cause neuroborreliosis, or most commonly in North America, to joint spaces, presenting as Lyme Arthritis (LA) (2, 3). LA is characterized by intermittent or persistent joint swelling and pain, primarily in large joints (4). Up to 60% of untreated LD patients are estimated to develop LA (3). Despite treatment with the appropriate duration of antibiotics, 10% of patients go on to develop Antibiotic Refractory Lyme Arthritis (ARLA), a form of Post-Treatment Lyme Disease Syndrome (PTLDS). The patient may require additional antibiotic therapy, immunomodulatory treatments, or even surgical intervention (5, 6). There is a substantial knowledge gap regarding the immune response of synovium to Bb once in the joint space. Further understanding of the acute immunomodulatory response of the joint space may be essential for the development of new treatments.

Resident synovial macrophages (RSMs) have been identified as key in joint space responses to inflammation (7, 8). RSMs are locally-renewing, M2/anti-inflammatory cells, residing within the synovial intimal lining (IntL) (7). RSMs restrict entry of cells into synovial fluid through tight junction connections, creating a proposed barrier structure surrounding the joint space (7). This barrier exists in both homeostatic and pathologic conditions, with increased cellular turnover in infectious and inflammatory conditions (9). Increased turnover was also associated with more severe patient symptoms. Further, RSMs may become M1/inflammatory in active rheumatoid arthritis (RA), indicating changes in functional polarity also coincide with loss of tight junction connections and an increase of circulating immune cells in synovial fluid (8). This points directly toward how alterations in RSM function and tight junction connection may alter patient symptoms in joint space disease beyond RA, including infectious arthritis such as LA (7–9).

To evaluate IntL responses that accompany Bb exposure and may relate to disease initiation and/or perpetuation, we infected fresh, intra-operatively retrieved human synovium with highly arthrogenic Bb wild-type strain B31 (OspC Type A) using our Joint Space Analysis System (JSAS) (10). This system maintains intact synovial architecture, allowing us to query the IntL and sub-lining (SubL) structures and inflammatory responses to Bb exposure. In this study, we asked three questions: 1) How does acute Bb exposure affect synovial barrier integrity? 2) How does Bb exposure alter synovial macrophage polarization and function? 3) How does Bb exposure modify the intrinsic secretome of the synovium? We found acute loss of IntL integrity on Day 2 that was maintained through Day 3. Rather than an M1-predominant profile, a mixed phenotype was observed—IntL macrophages maintained CD206 expression with a concomitant elevation of matrix metalloproteinase 9 (MMP-9) and inducible nitric oxide synthase (iNOS). There was minimal evidence of increased osteoclastogenesis. Finally, sustained IL-10 production from synovium, above constitutive levels, was observed with Bb exposure. These immune responsive changes from RSM are consistent with the natural course of LA as a cartilage-sparing, septic arthritis characterized by relapsing-remitting synovitis.

## 2 Materials and methods

### 2.1 Patient recruitment and synovial tissue processing

These studies were approved by the Institutional Review Board (IRB) at the University of Iowa, Iowa City, IA. Patients aged 18–80 years were included in the study if they were scheduled for non-emergent total joint replacement (hip or knee). Patients were excluded from the study if they had a history of septic arthritis on the operated joint, autoimmune arthritis, and/or were on systemic immunosuppressive medications. Patient demographics are shown in **Table 1**.

**Table 1:** Patient demographics.

Demographics		n=7
Age		(years)
	Mean	56.57
	Median	57
	Range	40-67
Sex		n, (%)
	Male	1, (14.3)
	Female	6, (85.7)
Joint		n, (%)
	Knee	6, (85.7)
	Hip	1, (14.3)

Excess anterior synovium (**Supplementary Figure 1A**) was excised from each patient and returned to the laboratory on ice, where it was washed twice in cold PBS. After careful dissection of excess adipose tissue, 3 mm biopsy cores (Integra) (**Supplementary Figure 1B**) were obtained, containing synovial IntL and SubL. Cores were placed in the Joint Space Analysis System (JSAS, **Supplementary Figure 1C**) (10). Briefly, a 24-well transwell system was established using 0.4 µm transwell inserts (Millipore Sigma) to create a two-chamber system. Six hundred µL of a 1:1 solution of Barbour-Stoenner-Kelly (BSK) H and low glucose Dulbecco’s Modified Eagle Medium (DMEM, Gibco) with 10% Fetal Bovine Serum (FBS) was added to the bottom chamber. Three hundred µL of low glucose DMEM with 10% FBS was added to the top chamber (**Supplementary Figure 1C**). One 3 mm biopsy core was placed in the top chamber and was completely submerged in media. No antibiotics were used. There were two technical replicates per treatment condition per time point, for each patient.

### 2.2 *Borrelia burgdorferi* culture and treatment

Highly pathogenic Bb, wild-type Strain B31, OspC Type A (ATCC 35210) was thawed from glycerol stock and cultivated in 10 mL BSK-H media at 34°C (11). When the culture reached late exponential growth phase at approximately 7–10 days, a Petroff-Hausser chamber was used to count Bb. The bottom chamber of each well of JSAS was inoculated with 250,000 (250k), 500,000 (500k), or 1,000,000 (1000k) Bb per well. Multiplicity of infection (MOI) could not be calculated due to intact, three-dimensional structure of synovium but is estimated at 1:600, 1:300, and 1:150 Bb:cell respectively (10). Lipopolysaccharide (LPS, Sigma-Aldrich) at 250 µg/mL was used as a known positive control for IL-1β and TNF-α activation in immunofluorescence studies. Plates were incubated at 37°C for 24 (Day1), 48 (Day 2), or 72 hours (Day 3) (**Supplementary Figure 1D**). Tissue samples were formalin fixed and paraffin-embedded (FFPE). Bottom well media was pelleted at 5000 x g for 10 minutes and supernatant was stored for ELISA.

### 2.3 Histomorphometry

**Figure 1:**
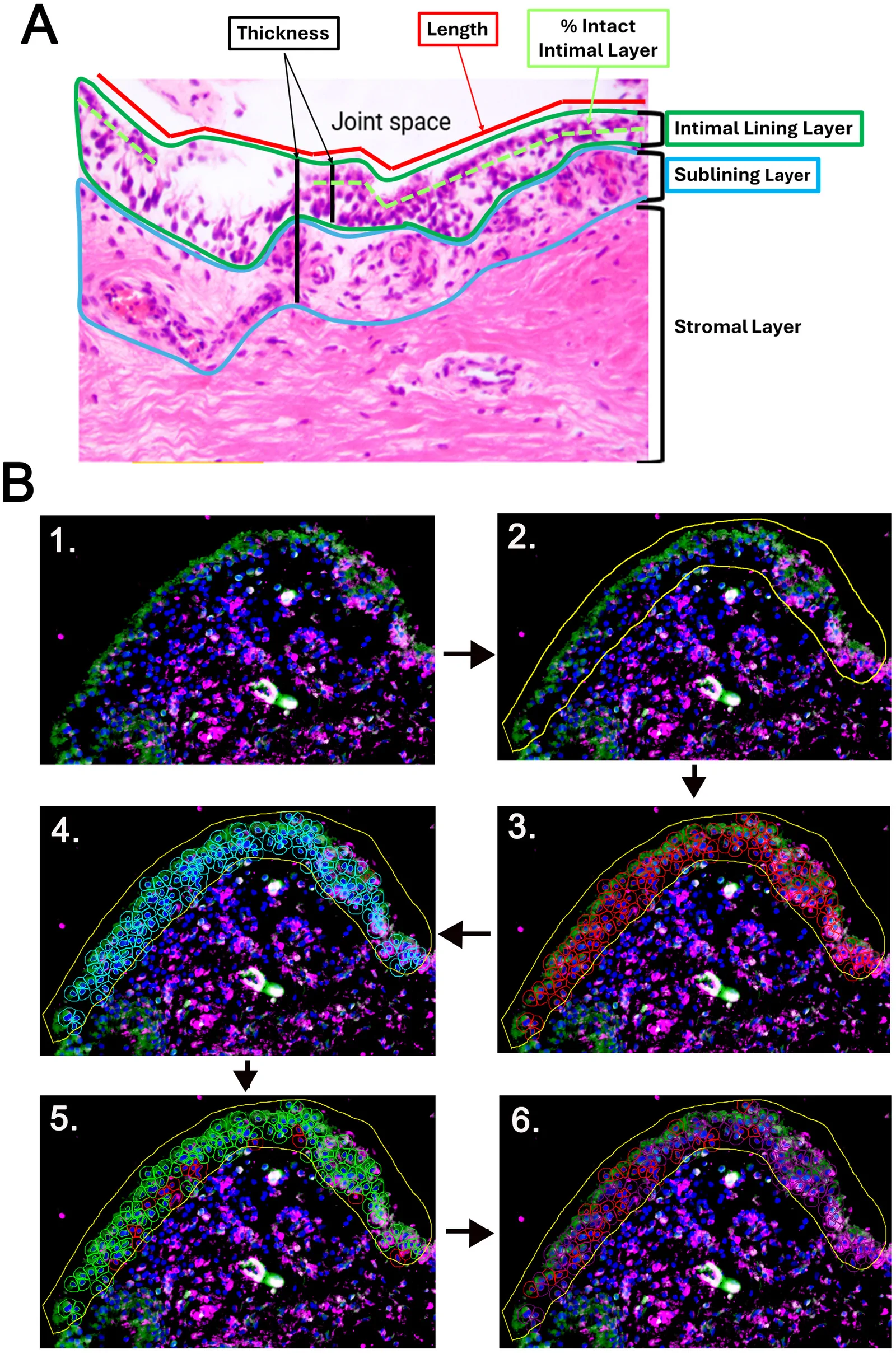
Illustrations to show how H&E and fluorescent images were analyzed using FIJI and QuPath. **(A)** An example H&E of synovium to identify normal structures. Dark Green outline – IntL bordering joint space. Blue outline – SubL containing synovial fibroblasts and macrophages. Thickness (black) of layers, length (red) of layers, and % intact IntL (light green) was measured using FIJI. **(B)** Flow Chart illustrating how QuPath was used to quantify the number of cells containing a particular fluorescent signal. 1. Fluorescent image before quantification. 2. Isolating region of interest – IntL of the synovium (yellow outline). 3. Quantification of all cells (red) within region of interest. 4. Quantification of all DAPI+ cells (blue). 5. Quantification of all CD68+ cells (green). 6. Quantification of all iNOS+(ex.) cells (purple). All double-positive and Triple-positive cells were quantified in the same manner.

Microscope slides containing serial sections of FFPE synovial tissue were stained with Hematoxylin and Eosin (H&E). Three representative 10X and 10 representative 40X brightfield images were taken per experimental endpoint across 2 technical replicates. Images were analyzed for IntL thickness (μm), IntL+SubL plumb depth thickness (μm), IntL cellularity (cells/100 μm), SubL cellularity (cells/100 mm^2^), and IntL integrity (intact IntL length/total IntL length). To calculate these measurements, Adobe Photoshop (v26.11.2) was used to create masks, isolating IntL or SubL within the image (**Figure 1A**). ImageJ/FIJI (v.1.54p) was used to collect measurements from each image using Measure and Analyze Particles feature. Specifics regarding mask creation and measurements were published previously (10). All measurements were averaged per patient when used in statistical analysis.

### 2.4 Secretome analysis

Sandwich Enzyme-linked Immunosorbent Assays (ELISA) included TIMP-1 (Biomatik, 1:10-1:20 dilution) and sRANKL (MyBioSource, 1:1 dilution). Each ELISA was performed per the manufacturer’s protocol. For multiplex ELISA, a custom 8-plex Human Magnetic Bead multiplex panel (Millipore Sigma) was used to analyze the concentrations of INF-γ, IL-1β, IL-4, IL-10, TNF-α, Osteoprotegerin (OPG/TNFRSF11B), Tartrate Resistant Acid Phosphatase (TRAP-5), and MMP-9. Multiplex was performed per manufacturer’s protocol. Median Fluorescence Intensity (MFI) was measured using a Luminex Bio-Plex 200 (Bio-Rad) instrument according to manufacturer’s guidelines.

### 2.5 Immunofluorescence

FFPE sections were deparaffinized, heat treated at 95°C in sodium saline citrate for 10 minutes and then blocked with 5% goat serum and 0.4% Tween-20 in PBS for 30 minutes at room temperature. Primary antibodies used were against CD206 (Thermo Scientific MA5-32498, 1:1000), iNOS (Thermo Scientific PA1-036, 1:100), MMP-9 (Thermo Scientific MA5-32705, 1:100), IL-1β (Invitrogen P420B, 1:100), TNF-α (Invitrogen PA5-19810, 1:250), IL-10 (Bioss bs-0698R 1:100), OPG (Invitrogen MA5-151715, 1:1000), MERTK (Invitrogen MA5-53212, 1:1000), CD68 (Invitrogen 14-0681-82, 1:1000), Ki-67 (eBioscience 14-5698-82, 1:200), Cleaved Caspase-3 (Cell Signaling 9661S, 1:500), Cleaved Gasdermin D (Cell Signaling 10137S, 1:1000), Ninjurin-1 (Santa Cruz Biotechnology 136295, 1:250). Secondary antibodies Cy3 Goat anti-rabbit (Invitrogen, A10520), FITC Goat anti-mouse (Invitrogen, 31569), were used at 1:1000. DAPI was used for nuclear labeling at 1:1000. Imaging was performed using Olympus BX-63 Fluorescent Light Microscope with CellSens Dimension Acquisition and Analysis (v. 4.2) software. Three to five representative 10X images and ten 20X images were taken per experimental end point. Image analysis was done using OlyVIA (v. 2.6) and QuPath (v. 0.5.1.) as shown in **Figure 1B**. Briefly, a line was drawn around the IntL to isolate the region of interest (ROI). ‘Analyze’ tools were used to identify cells, DAPI+ cells, and further double or triple positive cells within the ROI. MFI was not utilized, only a binary positive or negative signal based on same batch no-primary-antibody/secondary-only controls. All measurements were included in the statistical analysis to understand the potential focal changes that occur within the synovium upon exposure to Bb.

### 2.6 RNAscope with paired IF

Manual RNAscope Multiplex Fluorescent Assay (v.2, Advanced Cell Diagnostics) was performed on FFPE sections according to manufacturer’s protocol. Briefly, tissue sections were deparaffinized and antigen retrieval was performed for 15 minutes at 95°C. Tissue sections were hybridized with B Bburg-RNA23S-C1 (Advanced Cell Diagnostics, 468211) at 40°C for two hours using the HybridEZ oven, followed by signal amplification. TSA Vivid 650 (Advanced Cell Diagnostics) was used for detection at 1:1500 dilution. RNAscope was immediately followed by IF with CD68, MMP-9, iNOS, and ZO-1 (Invitrogen, 61-7300, 1:1000). Imaging was performed with 10 representative 40X captures per slide. Co-location of Bb with ZO-1 in macrophages was performed using OlyVIA and QuPath as above.

### 2.7 Statistics

GraphPad Prism (v.10.6.1) was used for statistical analysis. For percentage-based analysis, zeroes (0) and ones (1) were adjusted using the Smithson & Verkuilen (12) calculation, then a logit transformation was applied and statistical tests performed. For proportion measurements (e.g. puncta/cell), a log transformation was performed before statistical analysis. Statistics on these figures represent the logit or log transformed data only. A two-way ANOVA with Tukey *post-hoc* analysis was used to analyze histomorphometry, IF signal, and RNAscope. Comparisons were performed within each individual treatment day according to respective control. Four Parameter Logistic Regression (myAssays.com) was used to analyze sandwich ELISA. Five-parameter logistic curve-fitting method was used to calculate analyte concentration for multiplex ELISA. For ELISA data, a Shapiro-Wilk test for normality was applied and then a two-way ANOVA with Tukey *post hoc* analysis was performed. Readings above maximum were listed at the maximum for calculations of significance. A Welch’s t-test was used to evaluate Bb versus no Bb for IF read-outs. Statistical significance was determined as p < 0.05.

## 3 Results

There were seven patients recruited for this study (**Table 1**). The age range of the patients was 40–67 years old, with a mean of 56.6 and a median of 57 years. Six out of seven patients were female. In six patients, the synovial tissue was taken from the knee, while in one patient the synovium was taken from the hip (e.g., pulvinar tissue). The hip tissue was obtained from a female patient.

### 3.1 Synovial structural changes are associated with Bb exposure

**Figure 2:**
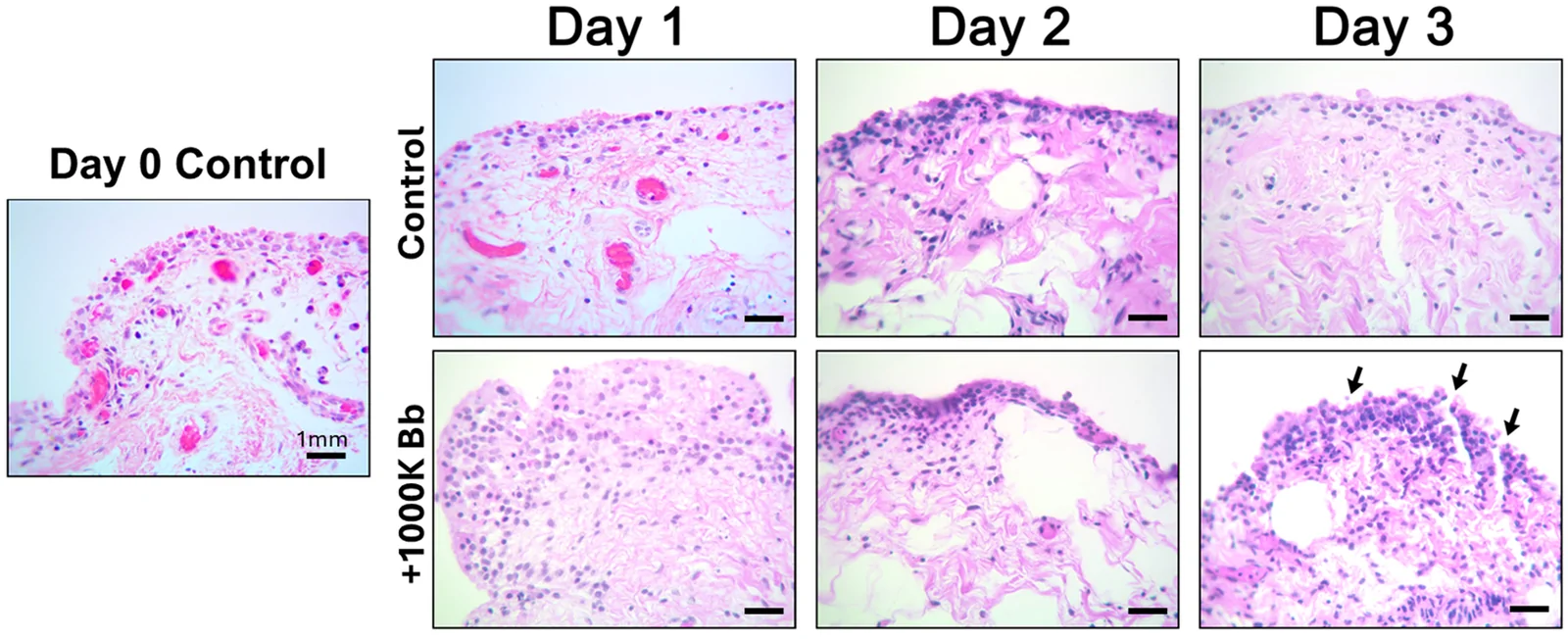
H&E of synovium after treatment with high dose Bb. Top images are control/untreated tissue. Bottom images are synovial tissue treated with 1000k Bb. Arrows point out breakdown of the synovial lining.

**Figure 3:**
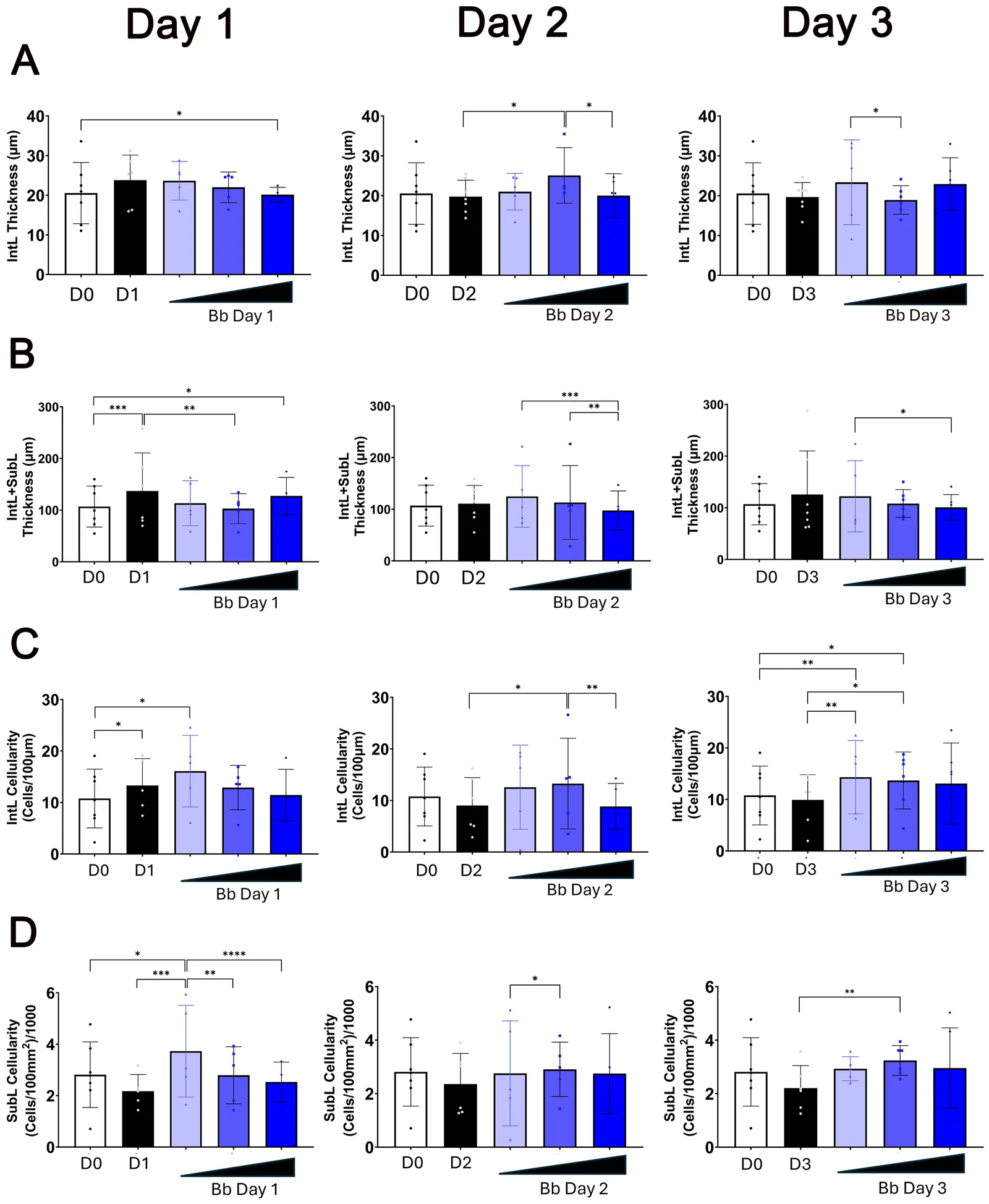
Quantification of synovial tissue stained with H&E. Thickness (μm) of IntL **(A)** and IntL+SubL **(B)** upon increasing Bb concentration at Day 1 (24hr), Day 2 (48hr), and Day 3 (72hr). Cellularity of IntL **(C)** and SubL **(D)** upon increasing Bb concentration at Day 1, Day 2, and Day 3. Increasing Bb concentration corresponds to 250k, 500k, 1000k Bb added to the JSAS Transwell System (10). Significance was calculated using 2-way ANOVA with Tukey *post-hoc* analysis. N = 4-7. Each data point = average of ten High Power Fields (HPF). *p-value less than 0.05; **p-value less than 0.01; ***p-value less than 0.001; ****p-value less than 0.0001. Error bars represent standard deviation.

Thickness of the IntL is characteristic of synovitis, while thinning may be indicative of fibrosis (13, 14), therefore we chose to investigate the thickness and cellularity of the IntL and SubL in response to Bb. The IntL and IntL+SubL of the synovium were visualized by H&E and measured using histomorphometry (**Figure 2**). IntL thickness was acutely and significantly decreased with 1000k Bb on Day 1 but increased with 500k Bb. By Day 3, these alterations had recovered and were no different than control (**Figure 3A**). There may be an observed dose-dependent, rolling, effect of thinning, thickening, and returning to baseline, occurring in the highest dose of Bb infection first, but the experimental time course was not sufficiently long to observe the changes of IntL in 250k Bb. Comparatively, when considering the much larger SubL (IntL + SubL plumb depth, **Figure 3B**), an acute, significant decrease in thickness was observed as early as Day 1 (500k) and continued through Day 3 (250k, 500k, 1000k) where thickness significantly decreased with increasing Bb concentrations. Changes in IntL thickness largely corresponded with modifications of IntL and IntL+SubL cellularity (**Figures 3C, D**; **Supplementary Figure 2**). However, cellularity seemed to recover quicker upon exposure to Bb, then IntL and SubL thickness. This suggests that the cellularity of the IntL and SubL primarily contributes to the thickness of the structures.

To determine if changes in cellularity were secondary to cell death and/or cell proliferation, we performed IF evaluating for cleaved Caspase-3 (cell death, apoptosis), Gasdermin-D (cell death, pyroptosis), Ninjurin-1 (cell death, pyroptosis), and Ki-67 (cell proliferation) in the Day 3,1000k Bb treatment group. We found high and focal expression of Ki-67 in IntL macrophages, suggesting recent replacement by interstitial renewing macrophages (**Supplementary Figure 3**). We also found expression of all three cell death markers especially in the SubL. This suggests that cell proliferation/cell death may be the main driver of cellularity modifications observed upon Bb exposure, and that dying cells are being actively replenished. More work must be done to evaluate the fibroblast component specifically.

**Figure 4:**
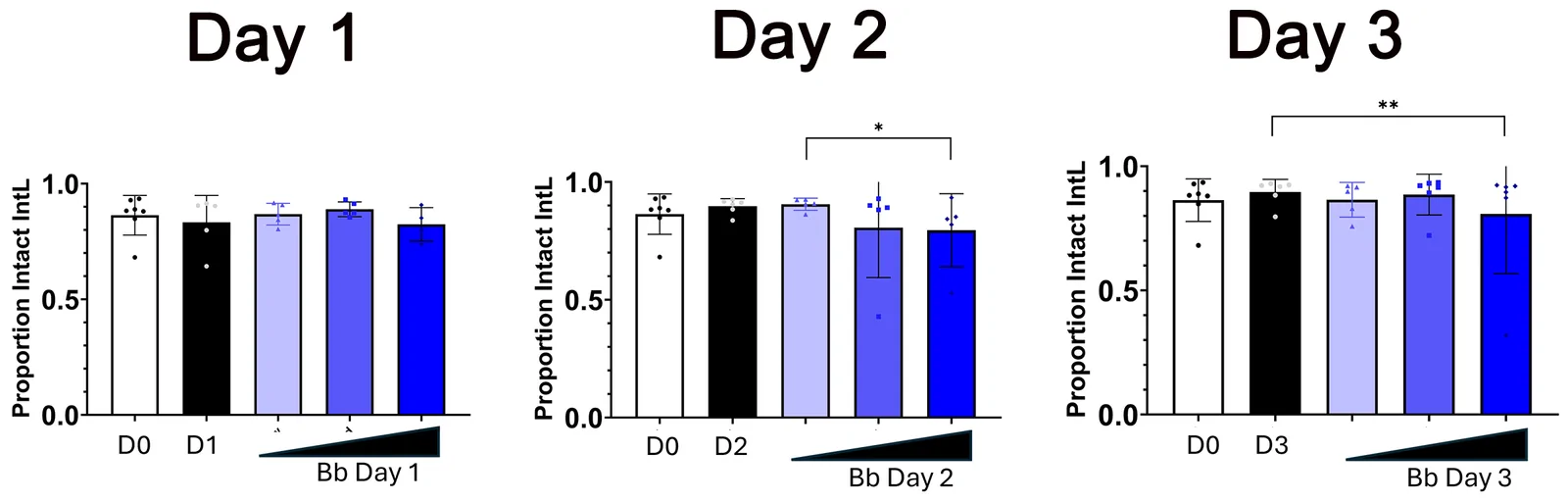
Quantification of intact IntL stained with H&E. The proportion of intact IntL border within the synovial tissue upon increasing Bb concentration at Day 1, Day 2, and Day 3. Increasing Bb concentration corresponds to 250k, 500k, 1000k Bb added to the JSAS Transwell System (10). Significance was calculated using 2-way ANOVA with Tukey *post-hoc* analysis after logit transformation. N = 4-7. Each data point = average of ten High Power Fields (HPF). *p-value less than 0.05; **p-value less than 0.01. Error bars represent standard deviation.

Barrier alterations within the synovium due to inflammation have been shown by 7, thus we hypothesized that the IntL border would be negatively affected by Bb exposure. IntL integrity was significantly decreased by 1000k Bb after Day 3 (p = 0.005) compared to same-day control (**Figure 4**). However, we observed a trending increase in intact IntL by Day 3 that suggests a potential preliminary recovery within the IntL border (**Supplementary Figure 4**). Overall, these observed synovial structural responses to Bb are consistent with the findings associated with acute synovitis and inflammation.

### 3.2 Bb exposure is accompanied by a joint-protective tissue response

**Figure 5:**
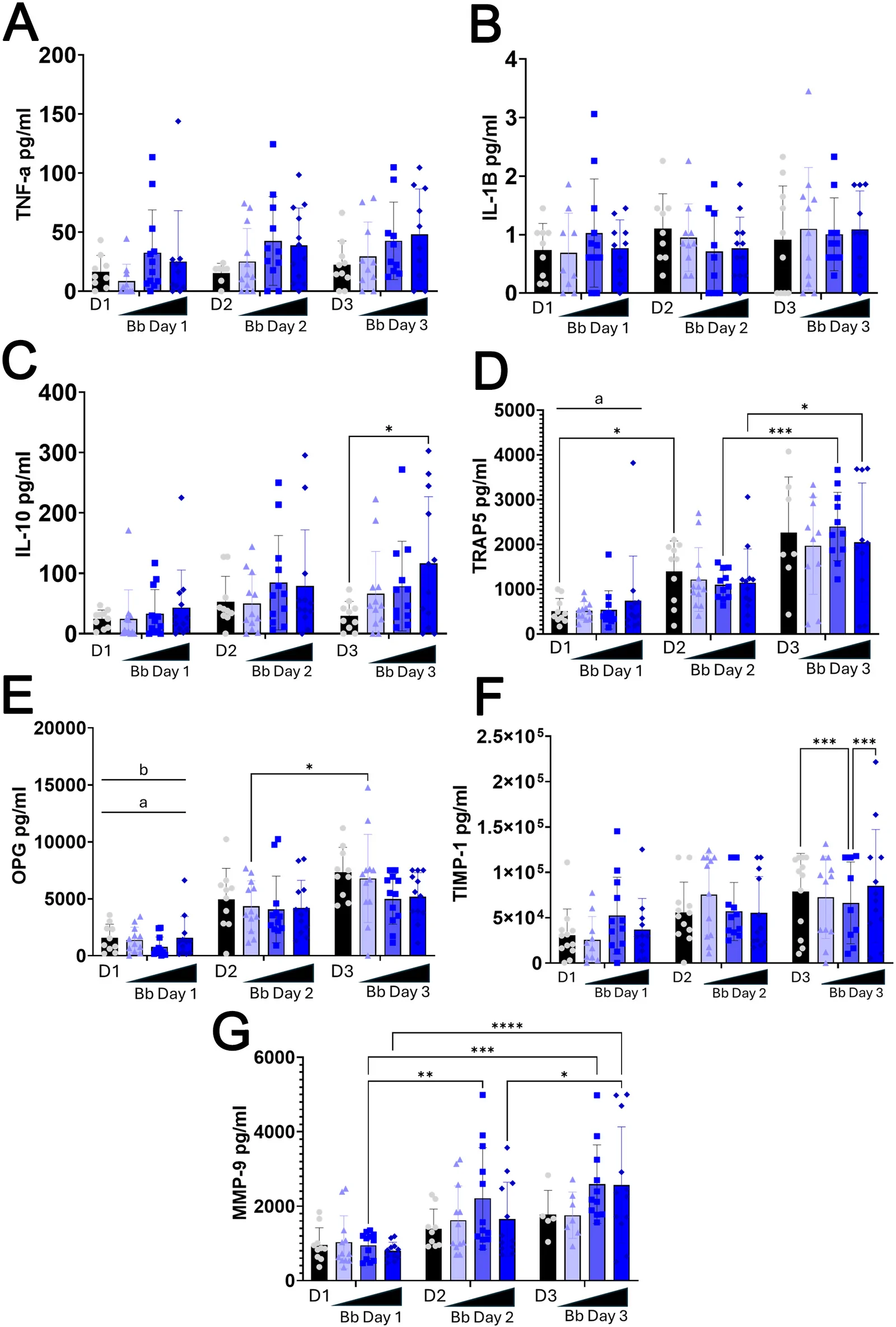
Enzyme-linked immunosorbent assays (ELISA) or custom 8-plex human magnetic bead multiplex. **(A)** TNF-α, **(B)** IL-1β, **(C)** IL-10, **(D)** Tartrate Resistant Acid Phosphatase (TRAP-5), a) Day 1 is significantly different from Day 3 (p < 0.0001 – 0.0005) **(E)** Osteoprotegerin (OPG), a) Day 1 is significantly different from Day 2 (p = 0.0018 – 0.0165), b) Day 1 is significantly different from Day 3 (p < 0.0001 – 0.0006) **(F)** TIMP-1, **(G)** MMP-9. Note: sRANKL, IFN-γ, and IL-4 were below the limits of detection (<1 pg/mL). Significance was calculated using 2-way ANOVA with Tukey *post-hoc* analysis. N = 5-6, each data point = 1 well replicate. *p-value less than 0.05; **p-value less than 0.01; ***p-value less than 0.001; ****p-value less than 0.0001. Error bars represent standard deviation.

Next, we examined several inflammation- and arthritis-related proteins for their possible secretion by the synovium in response to Bb. Secretome analysis showed that inflammatory cytokines TNF-α and IL-1β were produced in measurable quantities by the synovium, but there was no statistically significant increase by Day or Bb dose (**Figures 5A, B**). In addition, IFN-γ and anti-inflammatory type 2 cytokine IL-4, were tested but were below the limits of detection (<1 pg/ml, data not shown). A significant increase in the regulatory cytokine IL-10 was observed on Day 3 at 1000k Bb (**Figure 5C**).

Several arthritis-related proteins were found to have significantly altered expression in the presence of Bb with ELISA/Multiplex analysis. TRAP-5, a measure of osteoclast activity, was significantly increased by Day 3 (**Figure 5D**), however this seemed to be independent of Bb. OPG, the decoy receptor for the receptor-activator of NF-kB ligand (RANKL) and involved in preventing bone turnover, increased daily, independent of Bb exposure. However, at 500k and 1000k Bb, the increase from Day 2 to Day 3 was no longer significant, a pattern consistent with lower constitutive OPG secretion in Bb-exposed tissue (**Figure 5E**). Soluble RANKL (sRANKL) was tested but was below the limits of detection (<1 pg/mL, data not shown). TIMP-1 was highly expressed at all time points, while its target MMP-9 had increased secretion on Days 2 and 3. Regardless, TIMP-1 expression remained in excess in comparison (**Figures 5F, G**). Together, these data along with the increase in IL-10 release present an important secreted response by synovial cells to Bb.

### 3.3 With Bb exposure, OPG expression localizes to the IntL and IL-10 to the SubL

**Figure 6:**
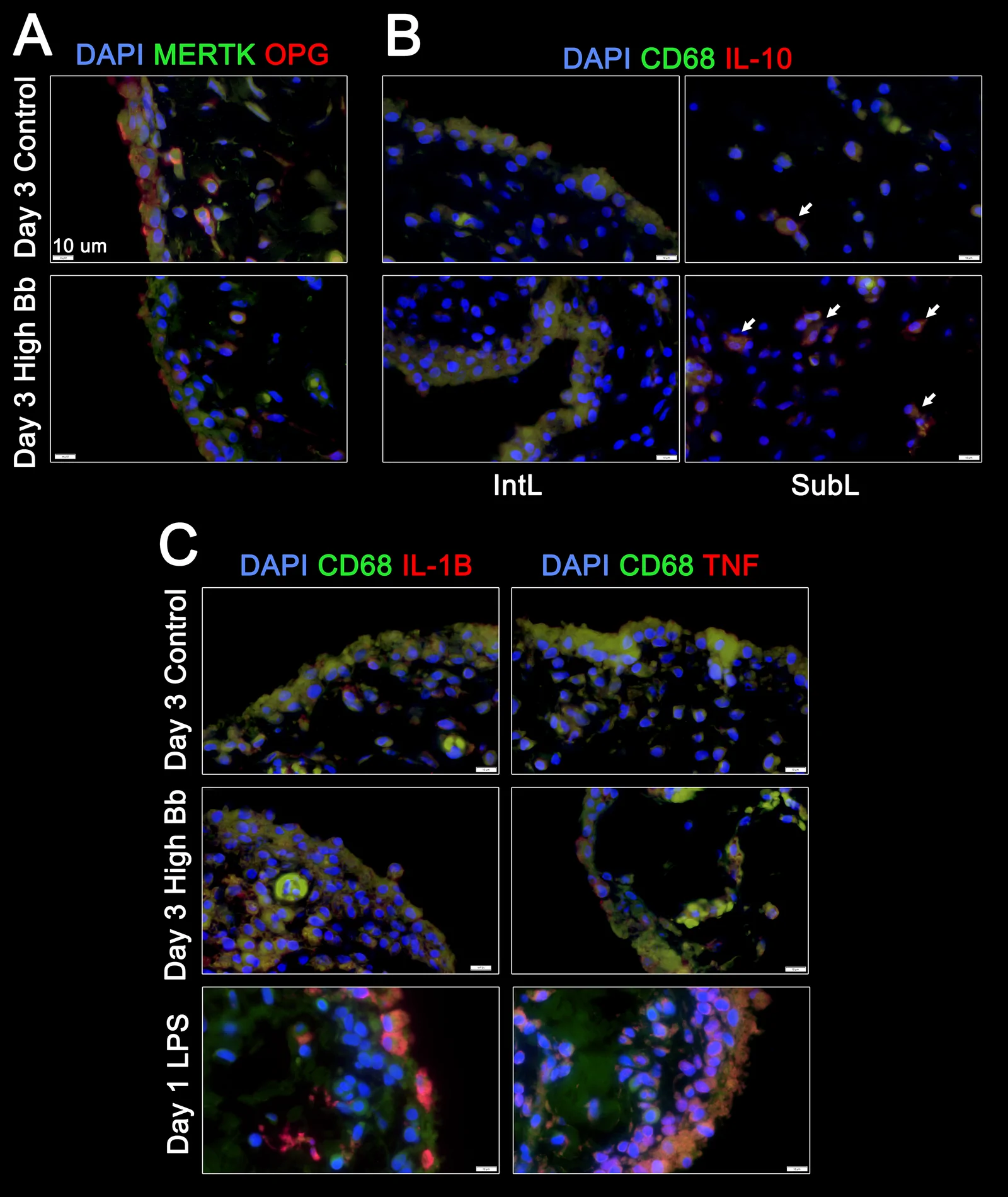
Immunofluorescence of OPG, IL-10, IL-1β, and TNF-α. **(A)** OPG (red) at Day 3 without Bb (Control) and at High (1000k) Bb. Cell nuclei were stained with DAPI (blue) and MERTK (green) was used as a marker for macrophages. **(B)** IL-10 at Day 3 without Bb (Control) and High (1000k) Bb in the IntL and the SubL. Arrows show where IL-10 is upregulated in the SubL. Cell nuclei were stained with DAPI (blue) and CD68 (green) was used as a marker for macrophages. **(C)** IL-1β and TNF-α at Day 3 without Bb (Control) and at High (1000k) Bb. As a positive control for cytokine upregulation, cells were treated with LPS for 24hrs (Day 1). Cell nuclei were stained with DAPI (blue) and CD68 (green) was used as a marker for macrophages. All images for the same protein/cytokine marker are of tissue from the same patient (images of LPS treated tissue are a separate patient).

Given the alteration in OPG and IL-10 secretion determined by Multiplex ELISA, we sought to determine where in the synovium these joint-protective cytokines were expressed. There was co-expression of IntL macrophage marker, MERTK and OPG, with apparent OPG down regulation with Bb exposure (**Figure 6A**). There are no reliable recorded instances of OPG and macrophage co-expression, so it is possible this represents FLS in the IntL. Conversely, IL-10 was not expressed in the IntL but rather was both expressed and induced in SubL macrophages (**Figure 6B**).

While we detected very low levels of TNF-α and IL-1β in supernatant, these cytokines are found in high levels in infected synovial fluid (15, 16). To assess this incongruity, we performed IF targeted to these cytokines and found minimal expression in tissue, suggesting these cytokines are not made in great quantity in response to Bb exposure by the synovium (**Figure 6C**). In comparison, LPS (positive control) quickly induced both cytokines, with IL-1β expressed largely by IntL macrophages and TNF-α by both IntL and SubL cells. Thus, Bb infection and/or exposure does not induce a strong IL-1β and TNF-α response by synovial macrophages in JSAS at this time course.

### 3.4 IntL macrophages are anti-inflammatory but pro-remodeling

**Figure 7:**
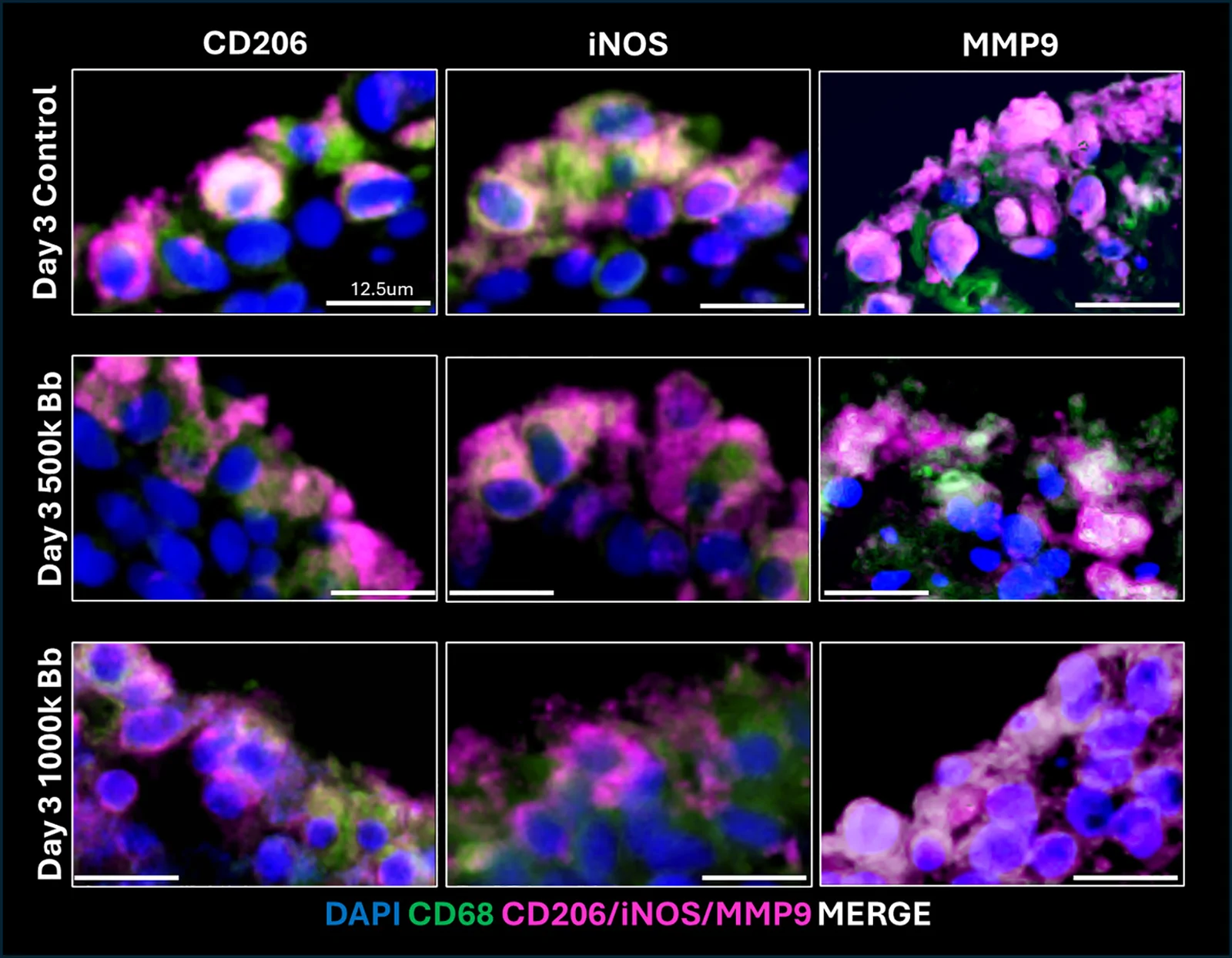
Immunofluorescence labeling of synovial tissue for various inflammation-related markers. Each image contains cell nuclei (DAPI, blue), macrophages (CD68, green), M2 macrophages (CD206, left panels, pink), inflammatory marker (iNOS, middle panels, pink), or matrix metalloproteinases (MMP-9, right panels, pink). White indicates co-localization of fluorescent signal. Top panels are control – no Bb treatment. Middle panels are after 72 hours (Day 3) at 500k Bb treatment. Bottom panels are after 72 hours (Day 3) at 1000k Bb treatment. All images for the same marker are from tissue from the same patient. Images were taken at 400X magnification using an Olympus BX-63.

**Figure 8:**
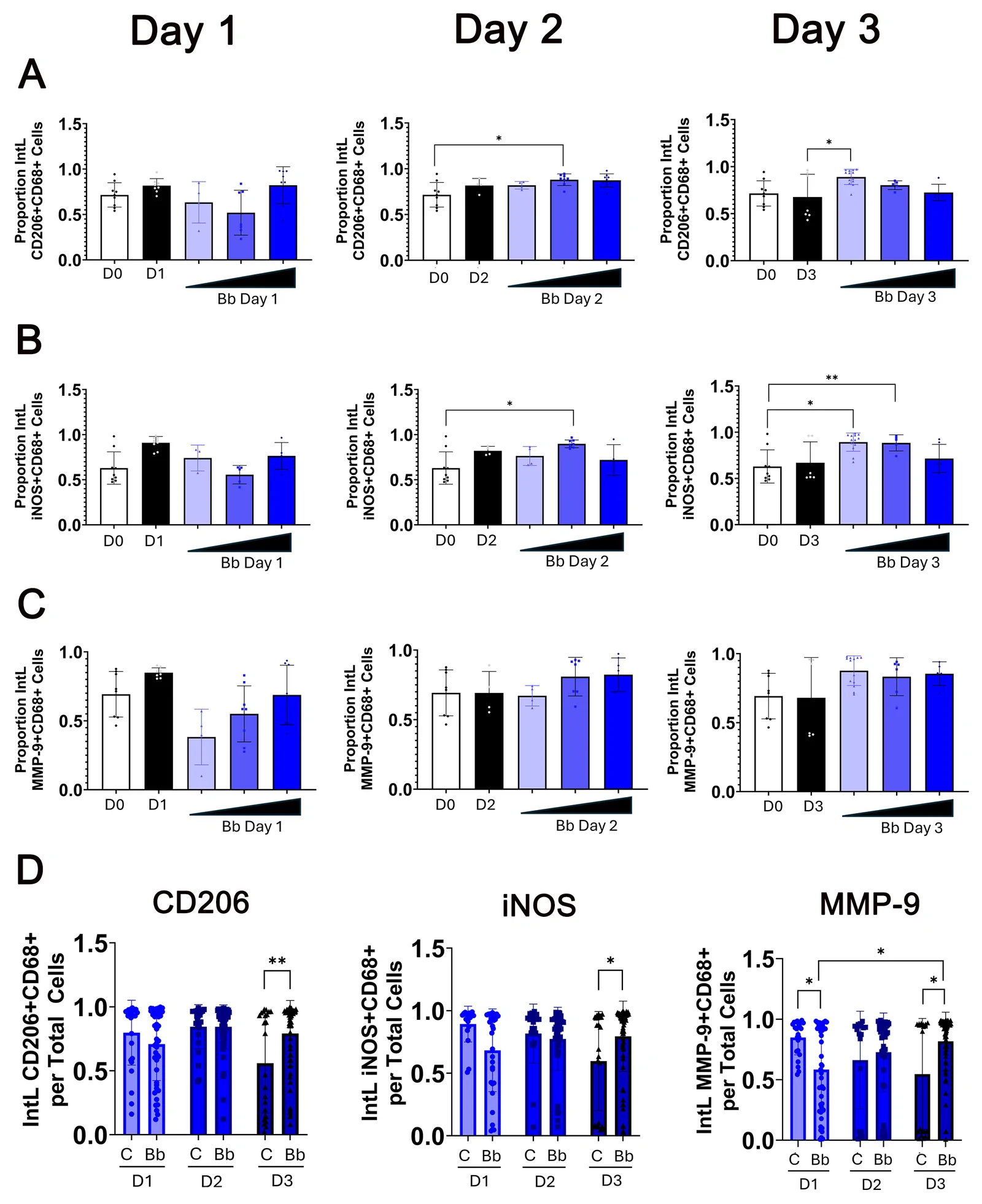
Quantification of immunofluorescence. Percent of DAPI-stained IntL cells that were Double Positive for CD68 and CD206 **(A)**, iNOS **(B)**, or MMP-9 **(C)** upon increasing concentration of Bb at Day 1, Day 2, and Day 3. QuPath was used to quantify the number of Double Positive cells. Significance was calculated using 2-way ANOVA with Tukey *post-hoc* analysis after logit transformation. N = 3-7; Each data point = 1 HPF. **(D)** Comparison of all Bb treated DAPI-stained IntL cells that were Double Positive for CD68 and CD206, iNOS, or MMP-9 versus Control (untreated cells, C) at Day 1 (D1), Day 2 (D2) and Day 3 (D3). Significance was calculated using 2-way ANOVA with Tukey *post-hoc* analysis after logit transformation. N = 3-7; Each data point = 1 HPF. *p-value less than 0.05; **p-value less than 0.01. Error bars represent standard deviation.

To assess how Bb exposure altered IntL macrophage polarization in intact tissue, we quantified DAPI+CD68+ co-expression with M2-marker CD206, M1-marker iNOS, and synovitis marker MMP-9 in IntL cells using QuPath. This data is expressed as a binary positive or negative, not MFI, based on 40X images (**Figures 1B**, **7**). Co-expression of CD206+ by IntL macrophages trended toward daily increases in 250k, but on Day 2 there was significant response with 500k Bb increasing compared to Day 0 controls, indicating an effect from JSAS culture. Dose-dependent responses reached significance only on Day 3 (250k) when compared to D3 control (**Figure 8A**). However, when compared by uninfected vs. infected, infected groups had a sustained increase in CD206 on Day 3 (**Figure 8D** CD206). Similarly, iNOS and MMP-9 expression had apparent alternations when compared to D0 controls as well (**Figures 8B, C**), but both were significantly increased on Day 3 when compared as uninfected vs. infected (**Figure 8D**). Together this suggests infection is associated with an IntL macrophage profile that sustains that sustains anti-inflammatory (CD206) activity, is pro-remodeling (MMP-9), and produces reactive nitrogen species (iNOS), but is likely underpowered to identify dose-dependent changes in this experimental design.

### 3.5 Altered RSM ZO-1 intracellular organization is associated with Bb exposure

**Figure 9:**
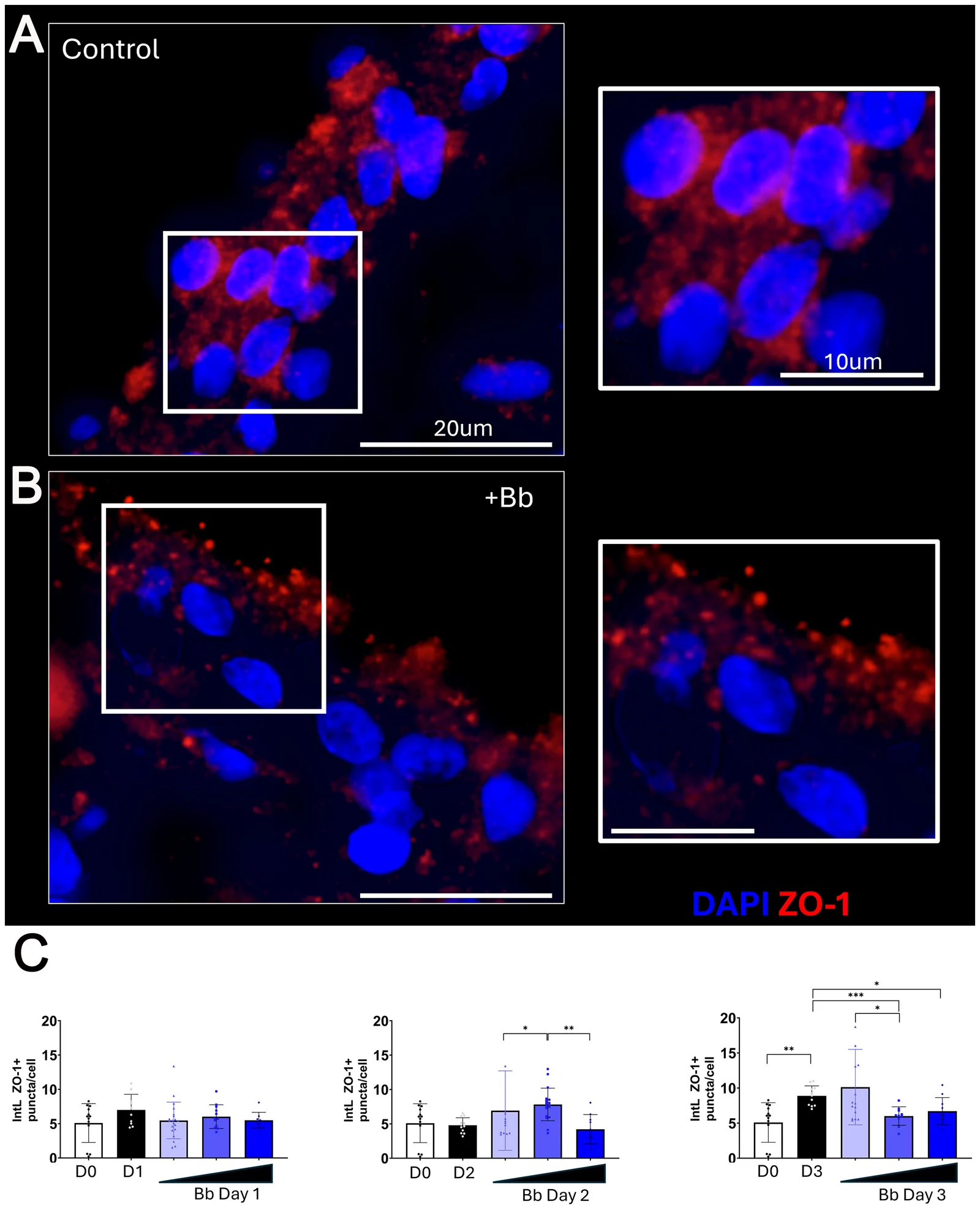
RNAscope paired with ZO-1 immunofluorescence. **(A)** synovium untreated (no Bb exposure). **(B)** synovium treated with a low dose (250k) Bb as a representative for Bb treated tissue. Each image contains tissue stained for DAPI (nuclei, blue) and ZO-1 (red). Images on left were taken at 400X magnification using an Olympus BX-63. Images on the right (inset) are zoomed in to better visualize ZO-1. Images are from the same patient after 3 days. **(C)** Number of ZO-1+ puncta per DAPI-stained IntL cell upon increasing concentration of Bb at Day 1, Day 2, and Day 3. Significance was calculated using 2-way ANOVA with Tukey *post-hoc* analysis after log transformation. N = 4-6; Each data point = 1 HPF. *p-value less than 0.05; **p-value less than 0.01; ***p-value less than 0.001. Error bars represent standard deviation.

Tight junctions are regions of semi-permeable paracellular barriers, facilitating near instantaneous cell-cell communication. Tight junction proteins such as ZO-1/TJP1 may provide important structure to the IntL and facilitate barrier formation between the synovium and joint space (7). ZO-1 proteins form cytoplasmic scaffolding that is critical for tight junction structure and function (17). On Day 0, ZO-1 is dense and peri-nuclear (**Figure 9A**). Upon treatment with Bb, ZO-1 becomes more punctate and diffusely distributed within IntL cells (**Figure 9B**), suggesting a reorganization of the tight junctions. Quantification of ZO-1 puncta identified a significant cellular decrease of ZO-1 puncta at 1000k Bb at Day 2 (p = 0.0065) and 500k and 1000k Bb at Day 3 (p = 0.001, p = 0.0484) (**Figure 9C**). This likely means that Bb exposure co-occurs with intracellular ZO-1 reorganization and ZO-1 downregulation, which together provide a possible rationale for loss of IntL integrity.

**Figure 10:**
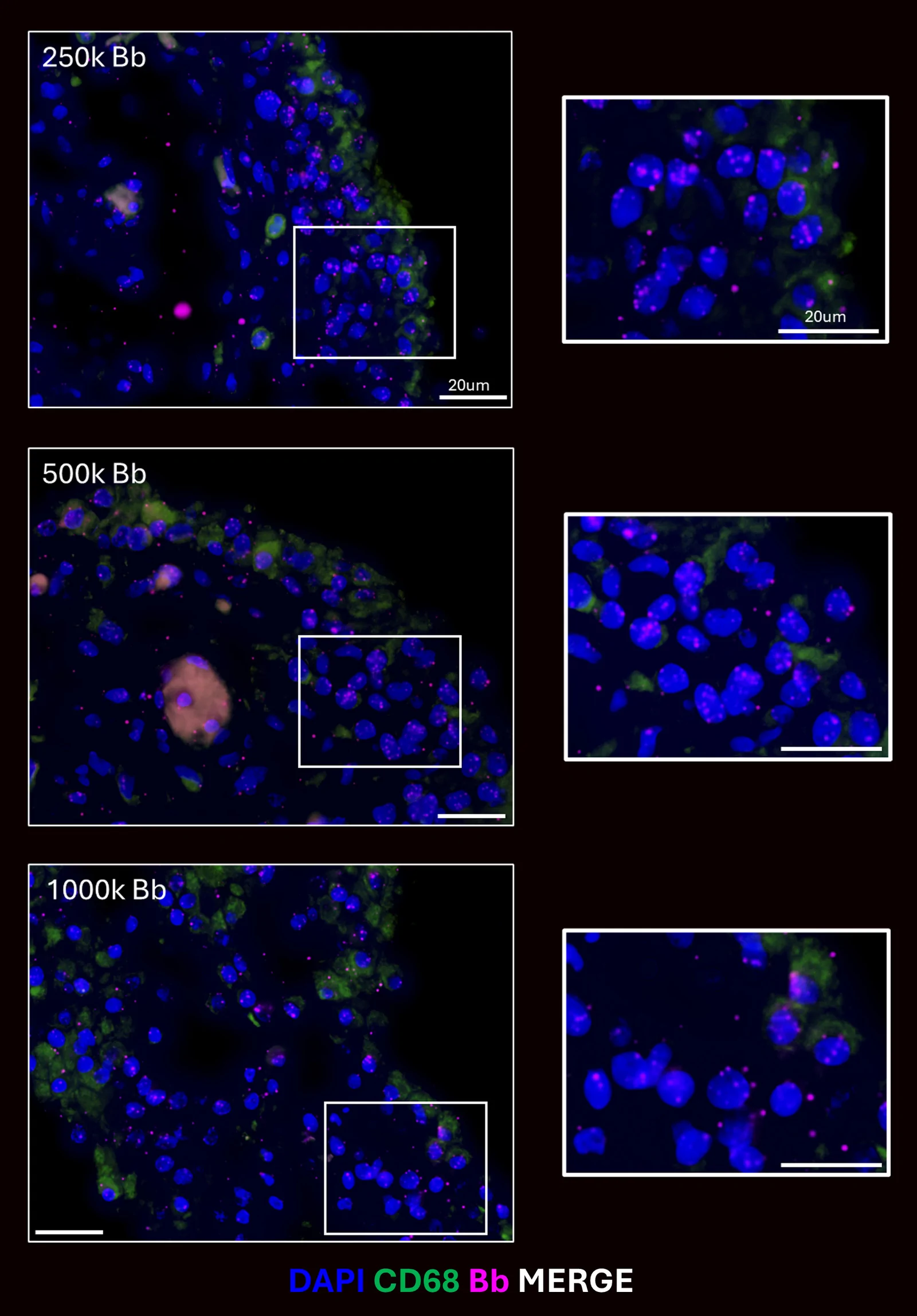
RNAscope paired with IF, Bb visualization. Top – synovium treated with a low dose (250k) of Bb. Center – synovium treated with a medium dose (500k) of Bb. Bottom – synovium treated with a high dose (1000k) Bb. Each image contains tissue stained for DAPI (nuclei, blue), CD68 (macrophages, green), Bb (pink). Images on left were taken at 400X magnification using an Olympus BX-63. Images on the right (inset) are zoomed in to better visualize the punctate dots of Bb. All images are from the same patient.

**Figure 11:**
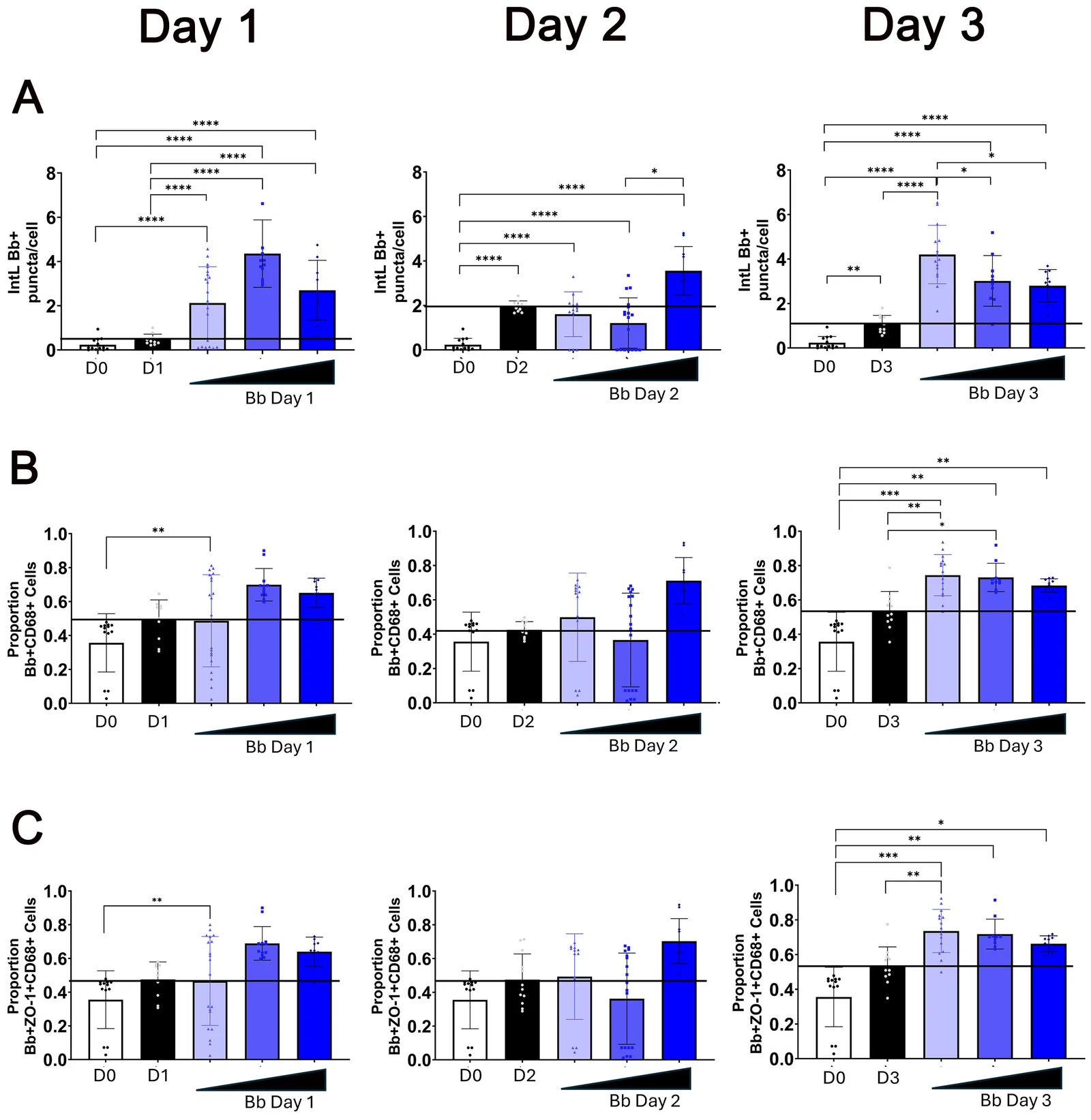
Quantification of RNAscope paired with IF for Bb. **(A)** Number of Bb puncta per DAPI-stained IntL cell upon increasing concentration of Bb at Day 1, Day 2, and Day 3. **(B)** Proportion of DAPI-stained IntL cells that were double positive for CD68 and Bb upon increasing concentration of Bb at Day 1, Day 2, and Day 3. **(C)** Proportion of DAPI-stained IntL cells that were triple positive for CD68, ZO-1, and Bb upon increasing concentration of Bb at Day 1, Day 2, and Day 3. QuPath was used to quantify the number of double/triple positive cells and the number of ZO-1 and Bb puncta/cell. Significance was calculated using 2-way ANOVA with Tukey *post-hoc* analysis after log transformation **(A)** or after logit transformation **(B, C)**. N = 4-6; Each data point = 1 HPF. *p-value less than 0.05; **p-value less than 0.01; ***p-value less than 0.001; ****p-value less than 0.0001. Horizontal black line represents false positive based on QuPath measurement in untreated control.

To confirm Bb infection inside synovial tissue and not paracrine effects to explain prior findings, we performed RNAscope targeted to the 23srRNA subunit of Bb. Signal was observed localized within IntL cells at all Bb concentrations and all three days of treatment, confirming tissue infection (**Figure 10**). This signal was highly focal and not widely dispersed across samples. Given our methodology was a positive or negative and not MFI, background was identified as false positive puncta. Thus, interpretation of IF data relies on same day control providing the cut off for signal: noise threshold: true positive Bb signal is only above the control and cannot be less than control. When evaluating the presence of Bb+ puncta in IntL regardless of cell type (**Figure 11A**), Bb signal was elevated on Day 1 in all treatment groups, globally unchanged from same day control on Day 2, and increased again on Day 3, possibly indicating migration of Bb within the tissue. Significant increases in identified IntL macrophages (CD68+) that co-stained with Bb were observed at Day 3 in 250k and 500k Bb treatments (**Figure 11B**), indicating possible infection or engulfment by macrophages specifically. When evaluating corresponding alterations in macrophages with Bb also expressing ZO-1, this recapitulates findings in **Figure 11B**, indicating these IntL macrophages are all likely RSMs expressing tight junctions, and that Bb signal co-localizes with this cell type in particular.

**Figure 12:**
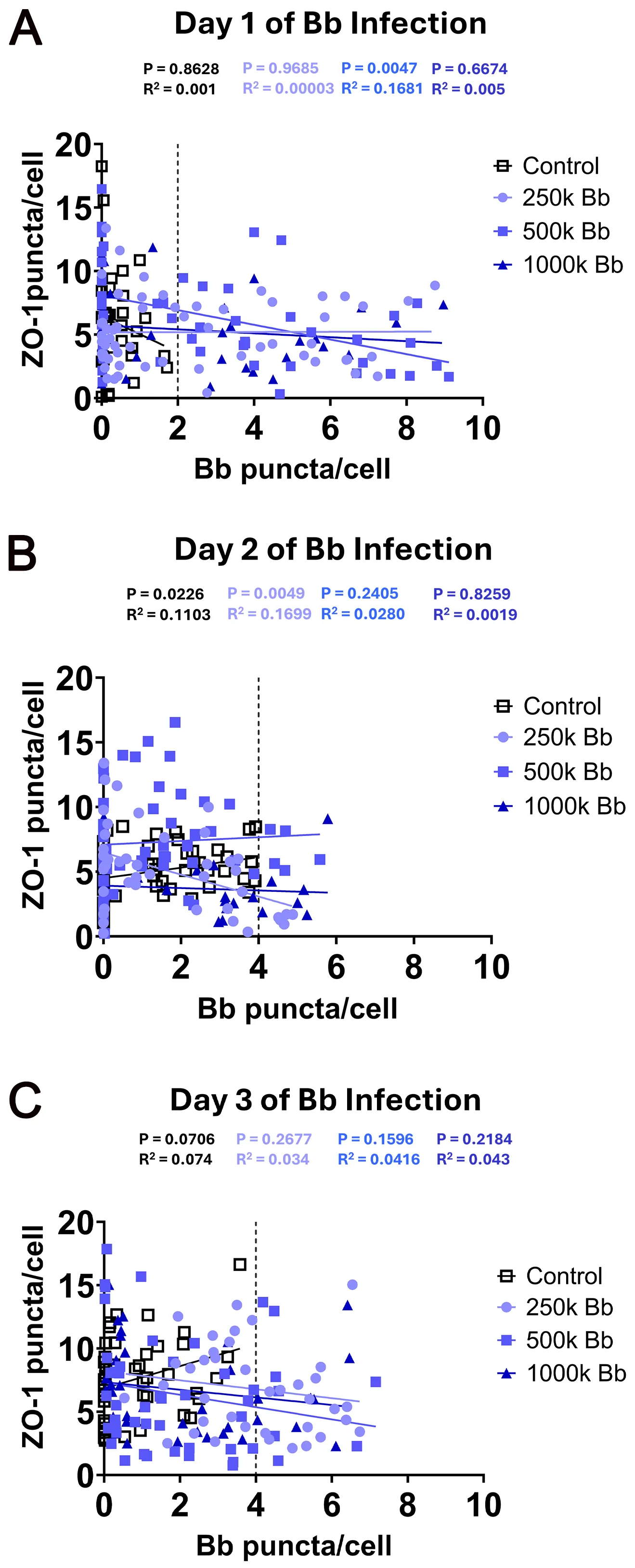
Linear regression of Bb puncta vs ZO-1 puncta with low, medium, and high Bb. **(A)** Bb puncta/cell vs ZO-1 puncta/cell linear regression on Day 1 of Bb exposure at Control, 250k Bb, 500k Bb, and 1000k Bb treatment. **(B)** Bb puncta/cell vs ZO-1 puncta/cell linear regression on Day 2 of Bb exposure at Control, 250k Bb, 500k Bb, and 1000k Bb treatment. **(C)** Bb puncta/cell vs ZO-1 puncta/cell linear regression on Day 3 of Bb exposure at Control, 250k Bb, 500k Bb, and 1000k Bb treatment. P-values and R-squared values were calculated by simple linear regression after log transformation. Lines with P-values <0.05 are considered significantly different from zero.

To determine how acute Bb exposure alters tight junctions and therefore possibly loss of IntL integrity, we performed linear regression of ZO-1 and Bb puncta. This showed a significant relationship at Day 1 with 500k concentration of Bb (p = 0.0047) and Day 2 with Control and 250k concentration of Bb (p = 0.0226 and p = 0.0049, respectively) (**Figures 12A, B**). However, these relationships had R^2^ values < 0.2, suggesting this alteration is minimally due to direct Bb presence and likely secondary or indirect inflammatory alterations and secretory effects in tissue with Bb exposure.

## 4 Discussion

**Figure 13:**
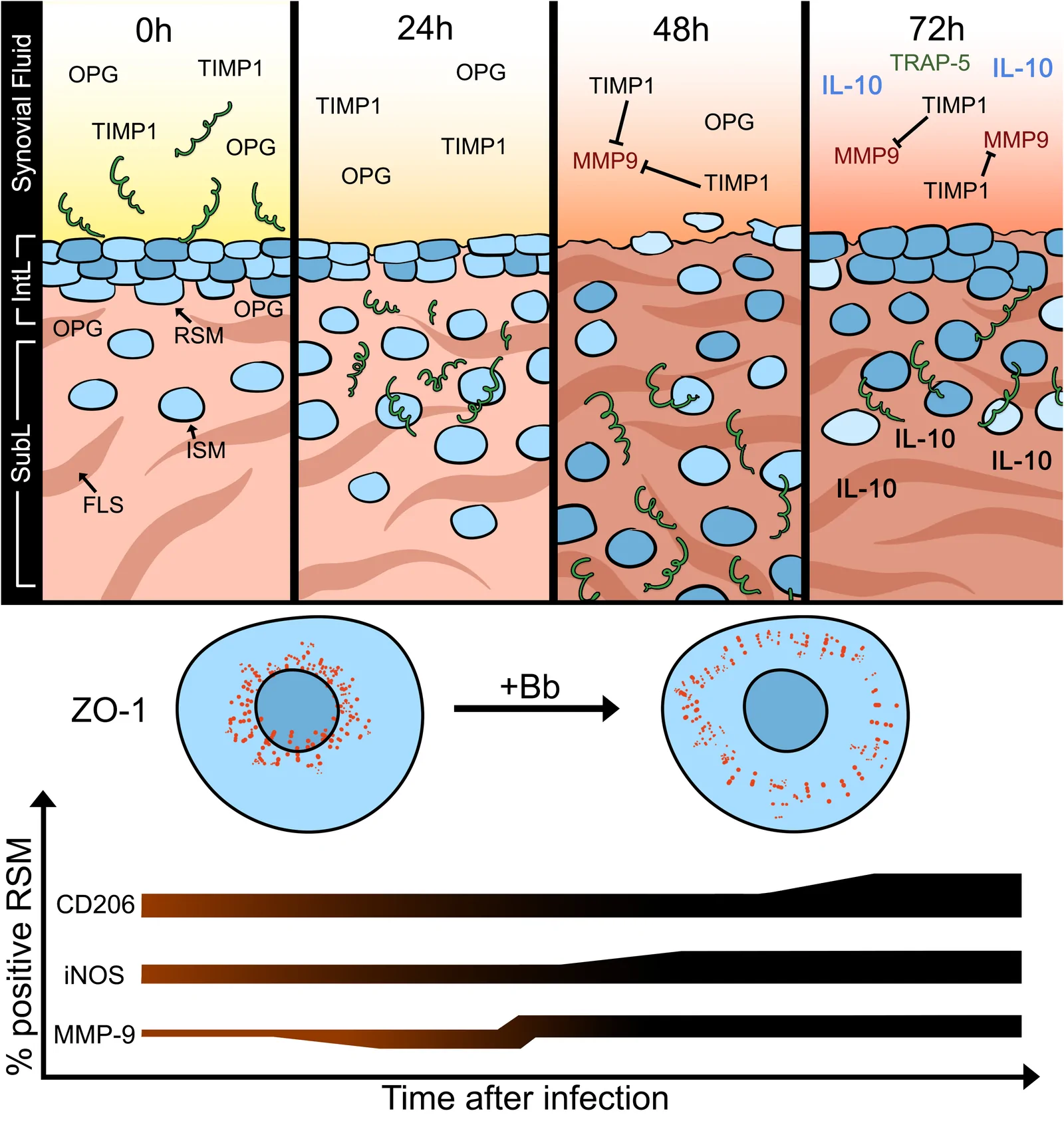
Synopsis of experimental findings. Under normal conditions (0hr), synovial fluid contains OPG and TIMP-1 that serve to protect the joint from cartilage damage. IntL is intact with a layer of RSMs held together with tight junction proteins. In the first 24hrs of acute Bb exposure, IntL begins to breakdown as ZO-1 tight junctions (red dots) are altered, and Bb enters the synovium. After 48hrs of Bb exposure, the IntL is completely broken down in local areas, Bb has penetrated through the IntL to the SubL of the synovium, and pro-inflammatory signals (MMP-9 and iNOS) are being expressed in the synovial tissue. Finally, 72hrs after Bb exposure the synovium begins to reestablish the IntL and recover. The IntL begins to rebuild, the regulatory cytokine, IL-10 is expressed at high levels in SubL and released at increased levels into the synovial fluid, and the anti-inflammatory marker CD206 is expressed at higher levels in the synovium. In addition, while MMP-9 remains high in the synovial fluid, TIMP-1 is expressed in excess and seems to prevent damage. TRAP-5 is secreted at high levels, while OPG secretion and tissue expression levels are downregulated.

In this work, we utilized a novel, *ex vivo* system and human synovial tissue to query how Bb alters early synovial lining macrophage responses in the acute phase of LA. We found evidence of rapid onset—but possibly cyclical—damage to synovial IntL in response to increased Bb exposure. This is consistent with the relapsing-remitting nature of LA symptoms (18). Steere and colleagues described an oligoarthritis with brief periods of mild to moderate pain, tenderness, and warmth. These symptoms repeated for up to 6 years and may be due in part to the synovial response to initial Bb exposure and/or tissue infection observed in this study. Further, we found the synovium intrinsic response to Bb was largely regulatory (e.g. increased IL-10, CD206), not inflammatory. This is consistent with mice studies that have shown that higher IL-10 levels are found in Lyme disease resistant mice and are correlated with lower production of inflammatory mediators (19). **Figure 13** presents a synopsis of findings.

Several arthritis-related proteins were secreted by the synovium, including TRAP-5, OPG, TIMP-1, and MMP-9. While OPG was downregulated in response to late Bb exposure, sRANKL was not expressed in this system. This would indicate the intrinsic synovial response is osteoprotective with constitutive OPG expression, and that sRANKL in synovial fluid is likely produced by cells extrinsic to synovium. Our prior work in synovial fluid indicated extremely high sRANKL in settings of infectious or inflammatory arthritis, and the main source may possibly be T cells (9). While osteoclast-related protein TRAP-5 was secreted by synovium, there was no altered expression by Bb, and expression appeared constitutive. However, migratory macrophages can multinucleate into osteoclasts, and this represents potential for increased bone turnover (20). Lastly, MMP-9 expression was elevated both in tissue and in supernatant and had increased secretion with Bb exposure. This would indicate a main driver of synovitis through ECM turnover. However, TIMP-1 remained in 1000-fold excess, indicating the synovial intrinsic response is to inhibit MMPs (21).

Regarding our three main questions, we present the following answers:

1. How does acute Bb exposure affect synovial barrier integrity?

An important part of this work was quantifying cellular-level structural alterations associated with synovitis and relating this to immune cell functionality. This granular level of detail has not been previously performed to link synovial histomorphometry with synovial immunobiology. Previous work has shown that during RA flares, synovial cell tight junctions rapidly disintegrate, and this alteration to synovial structure is strongly associated with joint pain and swelling (7). Likewise, synovial barrier breakdown measured by RSMs in synovial fluid was also associated with patient presentations to the emergency department for septic arthritis (9). However, the role of synovial barrier integrity has not been as well evaluated in LA as it has in autoimmune arthritis. As typical scoring systems (DAS and DAS28; 22, 23) cannot be utilized in JSAS due to absence of the peripheral immune system, we relied on fully quantitative rather than semi-quantitative or qualitative scoring methods to identify the earliest synovial structural changes in LA. Our study found that Bb has quick-onset (24 hours), quick-resolution (72 hours), focal synovitis as measured by IntL thickness, IntL+SubL thickness, IntL cellularity, SubL cellularity, and IntL integrity upon high concentrations of Bb exposure.

With this work, we demonstrated that Bb exposure was associated with loss of synovial barrier integrity by 24 hours with partial recovery of integrity (**Figure 4**; **Supplementary Figure 4**) through likely replacement by precursors as indicated by Ki-67 staining (**Supplementary Figure 3**). The loss of IntL integrity coincided with alterations to ZO-1 tight junction subcellular localization (perinuclear to cytoplasmic), and a decrease in ZO-1 protein levels within the IntL macrophages co-expressing Bb signal. This indicates that morphologic alterations to IntL and particularly loss of IntL integrity are likely due in part to changes in tight junction organization and/or Bb migration, both of which co-occurred with IntL cell death. This acute reaction to Bb exposure may help to explain the intermittent pain and swelling symptoms described for LA (4, 18). Linear regression data shows a relationship between ZO-1 puncta changes and intracellular Bb within the synovium at Day 1 and Day 2 that may be due to the immune response to Bb. It is unclear if this represents Bb within lysosomes from phagocytosis (24–26), or if Bb enters the cells through another mechanism. This is an area of future work.

2. How will Bb alter synovial macrophage polarization and function?

RSMs of the IntL are polarized M2 as determined by expression of TREM2, MERTK, CD206, and CD163 (27, 28). While the M1/M2 paradigm is an oversimplification of *in vivo* plasticity, it is a convenient way to contextualize findings. When histomorphometry data was paired with analysis of RSM polarization and function, Bb exposure resulted in sustained anti-inflammatory activity via CD206 and IL-10 expression. IL-10 has been shown to indirectly protect the joint by inhibiting Bb-induced IL-17 in mice during LA (29) which protects the joint from an inflammatory cascade; and inhibiting the synthesis and release of TNF-α (30) which blocks apoptosis of osteoblasts. In addition, IL-10 inhibits the production of inflammatory mediators within macrophages upon Bb exposure in mice (19, 31) suggesting an M2 phenotype. Importantly, IL-10 is essential for clinical remission within RA patients (32), indicating synovial production may be essential for symptom resolution in RA and likely therefore LA (and possibly, prevent the transition to ARLA). With the absence of TNF-α or IL-1β production, together these indicate acute Bb exposure of the synovium was accompanied by sustained anti-inflammatory activity via CD206 and IL-10. This is in the absence of the peripheral immune system and represents the intrinsic synovial response to Bb exposure.

This sustained regulatory phenotype is consistent with the unique host-pathogen interaction of Bb in humans. Unlike other bacteria that cause septic arthritis such as *staphylococcus* and *streptococcus* species, Bb does not produce proteolytic enzymes or toxins, except a potential neurotoxin (33, 34). This may explain why traditionally LA does not cause damage to articular surfaces through activation of inflammatory pathways compared to LPS-producing species (33, 35). Instead, we found that iNOS expression increased with Bb exposure. It is possible that induction of iNOS is an anti-microbial response. Inducible NOS is essential for production of reactive nitrogen species (RNS) such as nitric oxide (NO). While Bb DNA is not susceptible to damage by NO or reactive oxygen species (ROS) due to efficient DNA repair mechanisms (36), Bb proteins harboring free or zinc-bound cysteine thiols are targets of RNS (37, 38). However, increased NO directly results in decreased superoxide, diminishing oxidative burst capacity and anti-microbial lipid peroxidation (39). Further, paracrine NO signaling may be anti-inflammatory in OA (40). Conversely, others have shown that RNS have a role in inflammation and cartilage damage in both OA and RA (41). Thus, NO production may be pro-inflammatory, anti-inflammatory, anti-microbial, or all the above, and further work must be performed to evaluate the role of NO in LA.

While Bb may not produce a toxin to damage cells, in JSAS, MMP-9 expression in the IntL was elevated with Bb exposure. MMP-9 is associated with synovitis, ECM turnover, and peripheral macrophage migration and invasion in RA (42, 43). In LA, MMP-9 may also degrade or turn over the ECM in response to infection, alternatively, Bb hijacks MMPs to migrate through tissue as well (44). It is unclear if the observed elevation in MMP-9 is secondary to host immune response, Bb tissue migration, or both. As decreased MMP-9 correlated with thinning of the IntL, and increased MMP-9 with improved IntL integrity, MMP-9 production by the IntL may also be associated with IntL restoration by SubL renewing cells.

Similar polarization effects have been seen in evaluation of synovial fluid chemokine and cytokine levels in ARLA and antibiotic responsive LA. Patients with ARLA had significantly higher levels of pro-inflammatory chemokines and cytokines from Th1, macrophages, and neutrophils than LA patients responsive to antibiotics. This pro-inflammatory signaling continued even after antibiotic treatment in the ARLA patients (16). This suggests a loss of the regulatory immune environment in ARLA, which may result in extended and more severe symptoms. Indeed, this is supported by murine work where M2 macrophages dominated throughout Bb infection, with increased M1 macrophage levels only at the peak of inflammation (45). In human work in inflammatory arthritis and osteoarthritis, tissue resident macrophages within the synovium could transform between regulatory, pro-inflammatory, or anti-inflammatory depending on the stimulating factors (46, 47). Thus, it is possible that in LA, the potentially opposing markers expressed due to Bb exposure play balancing roles within the macrophage-populated IntL of the synovium.

3. How does Bb exposure modify the intrinsic secretome of the synovium?

We found that the synovium had constitutive expression of TRAP-5 and OPG. With Bb exposure, TRAP-5 secretion increased, which may indicate a pro-osteoclastic environment. Concurrently, OPG decreased, also indicating a shift toward bone and cartilage turnover. MMP-9 secretion was not measurably constitutive but was detected with Bb exposure. This indicates a distinct pro-arthritis environment, one which is counterbalanced by IL-10 induction and TIMP-1 excess that significantly increased with increased Bb exposure by Day 3. As LA does not typically result in either cartilage destruction or osteomyelitis, this secretome likely indicates a local—not broad—synovial response to Bb exposure.

TNF-α and IL-1β were not detected by ELISA, suggesting these cytokines are not produced at detectable levels by synovial cells with Bb exposure alone. However, in ARLA, TNF-α and IL-1β were found at higher levels within the synovial fluid and synovial tissue (16). In RA patients, both TNF-α and IL-1β are involved in the activation of RSMs to a pro-inflammatory phenotype (32). Together, these suggest that TNF-α and IL-1β may be expressed or induced by peripheral immune cells not present in our system. Alternatively, there may be individual variation in the induction of these highly inflammatory cytokines based on either genetic underpinnings or chronic exposure to antigen or pathogen. As we terminated our experiments at 3 days, chronicity could not be assessed. This is an area of future work.

### 4.1 Limitations

Although JSAS allows us to treat intact synovial tissue with external stimuli, several limitations must be acknowledged. First, the system lacks a peripheral immune system, which may be important given that dysregulation and dominant responses of T helper cells have been linked to ARLA (48–50), and Th17 cell plasticity has been closely related to disease activity in RA (51). However, JSAS allows us to understand the intrinsic immune response of synovium without outside signals. Second, the Bb exposure time course was limited to 3 days due to maximized tissue viability (10), making it difficult to observe the full immune response, which may be cyclical or result in permanent synovial changes with longer exposure. In the future, this can be assessed by adding peripheral blood mononuclear cells to the system in a systematical manner. Third, the tissue was obtained from patients with OA undergoing joint replacement. OA tissue is not “normal” and does not reflect what children, a major population affected by LD, experience. However, as LA is bimodal and affects older adults at similar levels as children (52), the tissue remains pertinent and accessible. In our patient population of primarily middle-aged women, the inflammatory effect may have been diminished compared to younger immune systems. Fourth, the Bb doses chosen were likely above physiologic infection levels, as *in vivo* Bb levels during infection are difficult to predict and measure, and synovial fluid taps would be under-representative since Bb can migrate to/from synovial tissue as shown here. Fifth, Bb sheds peptidoglycan continually, which may induce an immune response in the absence of active infection (53, 54). In our study, we did not differentiate between Bb glycoprotein exposure and tissue infection. This represents an important future direction where heat killed Bb or isolated glycoprotein could be used as external stimuli in JSAS to observe the synovial response. Sixth, although IntL barrier disruption was observed directly by immunohistochemistry and indirectly through immunofluorescence of ZO-1 tight junction protein, we did not determine if the function of the barrier was disrupted. Permeability assays to determine functional barrier disruption will require an *in vivo* model or extensive validation of our *ex vivo* model and represents important future work for this study. Seventh, we did not assess the causality of induced cytokines such as IL-10 or TIMP-1 to determine if they restore synovial function or structure as this is an important aspect of a dissertation with our laboratory and thus a key future direction. Finally, JSAS, including BSK-H media, represents a synthetic time course of Bb exposure that does not allow for the functional changes Bb undergoes when disseminated through blood *in vivo*, where gene and protein expression can be altered (55). Thus, it is unclear if the Bb used here would be representative of Bb gene expression from natural dissemination. To mitigate this, we used the B31 strain and infected with Bb from the late-exponential growth phase.

### 4.2 Conclusions

The work presented here indicates that the acute intrinsic synovial immune response to Bb exposure is transient and regulatory. Bb interaction with the joint lining co-occurs with rapid, structural and functional alterations in synovial tissue, with loss of intimal lining integrity and macrophage dysregulation that parallel the synovitis and joint swelling characteristic of LA. These alterations quickly recover back to near-normal even as Bb exposure and/or infection continues. The synovial barrier’s compromised state during infection may facilitate immune cell infiltration, contributing to disease pathogenesis, especially in the setting of increased markers of synovitis concomitantly with increased regulatory cytokines. Loss of the RSM regulatory phenotype whether via infiltrating immune cells or individual variation may result in ARLA, and this is an area of future study.

## Data Availability

Requests to access the datasets should be directed to KC, karen-cyndari@uiowa.edu.

## Supplementary material

Supplementary Figure 3 Immunofluorescence labeling synovial tissue for cell proliferation, apoptosis, and pyroptosis markers. Each image contains nuclei (DAPI, blue), macrophages (CD68, green or MERTK, pink). **(A)** Left panels: cell proliferation marker (Ki-67, red); Middle panels: apoptosis marker (Caspase-3, pink); Right panels: no primary antibodies added (only DAPI stain). **(B)** Left panels: pyroptosis marker (Gasdermin-D, pink); Middle panels: pyroptosis marker (Ninjurin -1, green); Right panels: no primary antibodies added (only DAPI stain). Each of the cell death/proliferation markers were used individually on serial sections of tissue. Top row is Day 0, no Bb exposure. Bottom row is Day 3 of 1000k Bb treatment. All images from the same treatment day are from the same tissue/same patient. Images were taken at 200X magnification using an Olympus BX-63.
